# Genetic Variation of *SAMM50* Is Not an Independent Risk Factor for Alcoholic Hepatocellular Carcinoma in Caucasian Patients

**DOI:** 10.3390/ijms232315353

**Published:** 2022-12-05

**Authors:** Hans Dieter Nischalke, Franziska Schmalz, Stephan Buch, Janett Fischer, Christine Möller, Madlen Matz-Soja, Benjamin Krämer, Bettina Langhans, Alexandra Klüners, Michael Soyka, Felix Stickel, Jacob Nattermann, Thomas Berg, Christian P. Strassburg, Philipp Lutz

**Affiliations:** 1 Department of Internal Medicine I, University Hospital, University of Bonn, 53127 Bonn, Germany; 2 Department of Medicine I, Dresden University Hospital, 01307 Dresden, Germany; 3 Division of Hepatology, Department of Medicine II, Leipzig University Medical Center, 04103 Leipzig, Germany; 4 Psychiatric Hospital, Ludwig Maximilians University, 80336 Munich, Germany; 5 Department of Gastroenterology and Hepatology, University Hospital of Zürich, 8091 Zürich, Switzerland

**Keywords:** cirrhosis, alcohol-associated liver disease, HCC, *SAMM50*

## Abstract

Hepatocellular carcinoma (HCC) is a severe complication of advanced alcoholic liver disease, which is modulated by genetic predisposition. Identifying new genetic loci might improve screening. Genetic variation of *SAMM50* was linked to HCC. We aimed to validate this finding in a large cohort of patients with advanced alcoholic liver disease (ALD). A large, well-characterised cohort of patients with alcoholic cirrhosis without (*n* = 674) and with (*n* = 386) HCC, as well as controls with HCC due to viral hepatitis (*n* = 134), controls with heavy alcohol abuse without liver disease (*n* = 266) and healthy subjects (*n* = 237), were genotyped for *SAMM50* rs3827385 and rs3761472 and for *PNPLA3* rs738409. Genotype frequencies were compared between patients with alcohol-associated cirrhosis with and without HCC by uni- and multivariate analysis. Minor variants in both *SAMM50* rs3827385 and rs3761472 were significantly more frequent in patients with alcoholic HCC versus alcoholic cirrhosis and versus the control cohorts. An even stronger association was noted for *PNPLA3* rs738409. The univariate analysis resulted in an odds ratio (OR) of 1.8 for carriers of at least one minor variant of *SAMM50* rs3827385 and rs3761472 (each *p* < 0.001), but this association was lost in multivariate analysis with age (OR 1.1/year), male sex (OR 3.2), diabetes (OR 1.9) and carriage of *PNPLA3* 148M (OR 2.1) remaining in the final model. Although minor variants of both *SAMM50* loci are strongly associated with alcoholic HCC, this association is not independent of carriage of the well-known risk variant *PNPLA3* 148M.

## 1. Introduction

Hepatocellular carcinoma (HCC), which is the term for primary liver cancer originating from hepatocytes, is the sixth most common cancer globally and the fourth most frequent reason for cancer-related death [1]. The prognosis of HCC is usually poor. Only when small tumours in a rather healthy liver are detected is the surgical removal of the tumour possible [2]. However, the tumour is often detected when curative treatment is no longer feasible. Therefore, early detection of HCC is supposed to lead to more efficient treatment and even cure [3]. Because HCC usually develops in patients with advanced fibrosis or cirrhosis, the target population for screening is rather well-defined [2]. However, given the shortage of health care providers, defining the patient subgroup at particular risk is necessary to reduce the number of patients to screen [4].

Alcohol-associated cirrhosis is a major risk factor for liver disease, ranging among the top three risk factors with some regional variations [2]. Alcohol abuse is estimated to account for 15–30% of HCC cases [1]. Because effective treatment options for hepatitis B and hepatitis C nowadays exist, the proportion of HCC arising from viral hepatitis is diminishing, while steatohepatitis increases as a cause for HCC [5,6], making this aetiology particularly important for screening programs. Once patients have developed chronic liver disease, the risk for HCC is strongly modulated by the presence of genetic risk factors [1]. Different genetic loci were discovered and validated in large patient cohorts, with some increasing the risk for HCC, such as the presence of the *PNPLA3* minor variant 148M [1,7,8], and with others reducing the risk, such as the *HSD17B13* rs72613567:TA variant [9,10]. Attempts were made to develop genetic risk profiles that may be used to select patients at particularly high risk [11]. The hitherto analysed risk scores in NASH [12,13], HCV cirrhosis [14] or alcohol-associated liver disease [15] also incorporate clinical data and improve risk stratification but still lack precision, which may be improved by the discovery of new genetic markers for HCC. However, differences between the ethnicities of patients and aetiologies of cirrhosis have to be taken into account.

New genetic markers that deserve validation are the minor variants at two polymorphisms in the *Sorting and assembly machinery component 50 homolog (SAMM50)*, rs3761472 T > C and rs3827385 A > G, which were identified in two large cohorts including 705 HCC cases and 1455 controls and were validated using several datasets [16]. Both polymorphisms are supposed to interfere with transcription factor binding, with *SAMM50* expression being increased in the presence of the minor variant in adipocytes [16]. Rs3761472 [17] and rs738491/rs2073082 [18] in *SAMM50* were associated with NAFLD before; another polymorphism in *SAMM50* (rs2143571) was linked to the serum level of ALT at the population level [19]. Therefore, *SAMM50* seems to be an interesting gene in relation to liver disease. However, in a study comprising a large multi-ethnic cohort, genetic variation in *SAMM50* was associated with NAFLD in Asians but not in Caucasian patients, hinting at a major impact of the ethnical background of the patients [20]. *SAMM50* is a mitochondrial protein crucial for the proper assembly of respiratory chain complexes [21] and is involved in the thermogenesis of adipocytes [22]. Since mitochondria are known to play a key role in the pathogenesis of fatty liver disease, genetic variation in *SAMM50* might be related to this process [23].

In the present study, we analysed if the *SAMM50* minor variants of rs3827385 and rs3761472, C and G, respectively, were linked to the occurrence of HCC in alcohol-associated liver disease to validate a potential association in a large European cohort including, among others, 386 HCC cases and 674 cirrhotic controls. 

## 2. Results

### 2.1. Study Population

Patients with cirrhosis with and without HCC had the typical age (mean ranging from 56.4 years in patients with alcohol-associated cirrhosis without HCC to 63.5 years in patients with alcohol-associated HCC), were as expected predominantly male (62–88%) and had a mean MELD score between 13.7 and 16.8. Laboratory liver parameters were slightly elevated and platelet count was decreased or at the lower end of the normal range. Details of the study cohorts are given in Table 1.

### 2.2. Genotype Distribution

Minor allele frequency (MAF) of *SAMM50* rs3827385 were 18.8% and 18.0% in healthy controls and controls with alcohol abuse but absent significant liver disease, respectively, which was comparable to the published MAF of 21.6% [24]. Similarly, the MAFs of *SAMM50* rs3761472 were 19.6% and 17.9%, respectively, in both control cohorts without liver disease, which corresponded to the published MAF of 18.8% [24]. The same held true for *PNPLA3* rs738409 with MAFs of 26.8% and 25.6%, respectively, in healthy controls and alcohol abusers without significant liver disease and 22.6% reported from the 1000 genome project [24].

The genotype distribution is shown in detail for each cohort and every polymorphism in Table 2. The heterozygous and homozygous minor allele genotypes of *SAMM50* rs3827385 and rs3761472 were more frequent in patients with alcohol-associated cirrhosis versus both control cohorts. This difference versus controls became more pronounced in patients with HCC on the background of alcoholic cirrhosis and became also statistically significant versus both control cohorts without liver disease, versus viral HCC and versus patients with cirrhosis (Table 2). An increased frequency of the heterozygous and homozygous genotype of the *PNPLA3* rs738409 minor allele was observed not only in patients with alcoholic HCC but was also statistically significant in patients with alcohol-associated cirrhosis versus both control groups without liver disease (Table 2). The same effect could be noted for both *SAMM50* minor variants but the homozygous minor variants failed statistical significance against both control groups without liver disease.

### 2.3. Frequency of the SAMM50 Minor Variants

**Figure 1:**
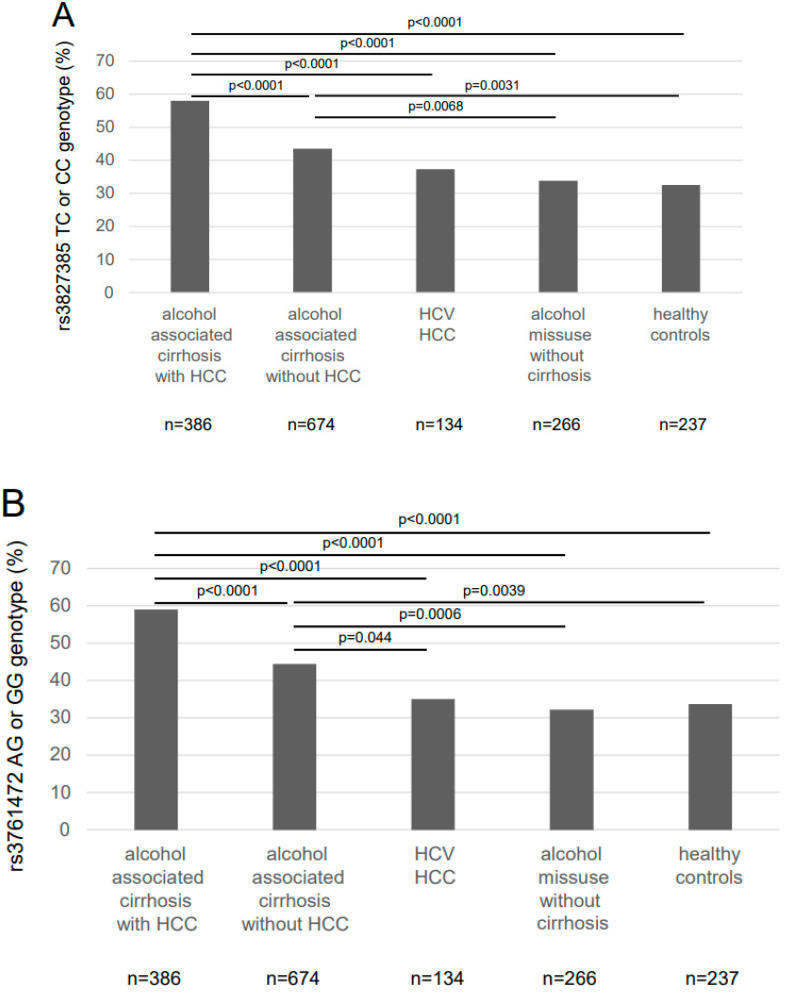
Proportion of carriers of a *SAMM50* minor allele among the study cohorts. Carriers of the minor allele are over-represented among patients with alcoholic hepatocellular carcinoma (HCC) versus all other cohorts and among patients with alcohol-associated cirrhosis versus controls without liver disease. (**A**) proportion of patients carrying a *SAMM50* rs3827385 genotype containing the minor allele C among the study cohorts. (**B**) proportion of patients carrying a *SAMM50* rs3761472 genotype containing the minor allele A among the study cohorts. Statistical analysis with Pearson’s goodness-of-fit Chi2 test. Abbreviations: HCC hepatocellular carcinoma; HCV hepatitis C virus; *SAMM50* Sorting and assembly machinery component 50 homolog.

When genotypes were not analysed separately but the carriage of the minor allele was evaluated, the patients with alcoholic HCC were 58.0%, patients with alcohol-associated cirrhosis were 43.5%, patients with HCC due to viral hepatitis were 37.4%, controls with alcohol abuse without liver disease were 33.9% and healthy controls were 32.5% carriers of the *SAMM50* rs3827385 minor variant (Figure 1A). Respective frequencies were 59.1%, 44.5%, 35.1%, 32.3% and 33.8% for the carriage of the *SAMM50* rs3761472 minor variant (Figure 1B). For both polymorphisms, carriers of a minor variant were significantly more frequent in the alcoholic HCC group versus the other cohorts; carriage of a minor variant was also more frequent for both polymorphisms in patients with alcohol-associated cirrhosis versus healthy controls or controls with alcohol abuse but no significant liver disease. Thus, both minor variants seemed to be risk factors for alcohol-associated cirrhosis as well as HCC.

### 2.4. Uni- and Multivariate Analysis for Various Risk Factors

Next, we performed a univariate analysis of several potential factors associated with the occurrence of HCC (Table 3), which showed that, as expected, age, male sex, diabetes, presence of a *PNPLA3* 148M allele and presence of a *SAMM50* rs3827385 minor allele or a *SAMM50* rs3761472 minor allele were associated with the presence of HCC. However, while the association with age per year (OR = 1.078 [95% CI 1.059–1.097]), male sex (OR = 3.174 [95% CI 2.081–4.480], diabetes (OR = 1.930 [95% CI 1.399–2.662] and the *PNPLA3* 148M risk variant (OR = 2.083 [95% CI 1.492–2.909] remained highly significant in multivariate analysis (Table 3), the association with each of the *SAMM50* minor alleles was lost when the regression model included the *PNPLA3* I148M risk variant, suggesting that variation of *SAMM50* rs3827385 and rs3761472 are linked to the risk factor *PNPLA3* 148M. 

### 2.5. Analysis of Linkage Disequilibrium between SAMM50 Variants and the PNPLA3 148M Variant

To test whether the three polymorphisms were in linkage disequilibrium, we calculated r^2^ and D’. For *SAMM50* rs3761472, D’ and r^2^ with *PNPLA3* rs738498 were 0.911 and 0.517, respectively; for *SAMM50* rs3827385 and *PNPLA3* rs738498 D’ and r^2^ were 0.799 and 0.391, respectively. For both *SAMM50* polymorphisms with each other, D’ and r^2^ were 0.875 and 0.751, respectively. In particular, the high D’ values underline the genetic linkage between these three polymorphisms.

**Figure 2:**
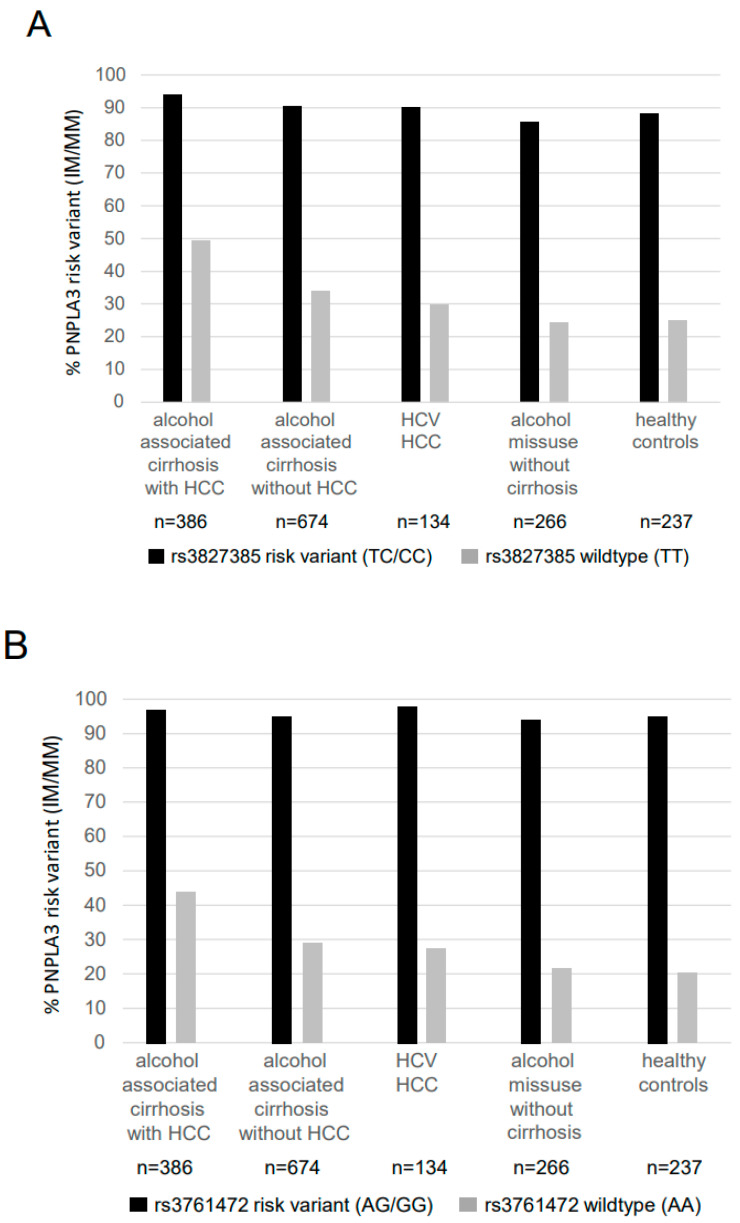
Proportion of patients carrying a *SAMM50* risk variant also carrying a *PNPLA3* 148M risk allele. This figure illustrates that the vast majority of patients carrying a *SAMM50* risk variant were also carriers of the *PNPLA3* 148M risk variant. Among carriers of a *SAMM50* wildtype, the frequency of the *PNPLA3* 148M risk variant increased in patients with alcoholic liver disease. (**A**) Displaying patients carrying a *SAMM50* rs3827385 TC/CC genotype (**B**) displaying patients carrying a *SAMM50* rs3761472 AG/GG genotype. Abbreviations: HCC hepatocellular carcinoma; HCV hepatitis C virus; *PNPLA3* patatin-like phospholipase domain-containing protein 3; *SAMM50* Sorting and assembly machinery component 50 homolog.

In addition, we analysed how many carriers of a *SAMM50* rs3827385 or rs3761472 risk variant were also carriers of a *PNPLA3* 148M risk variant. As shown in Figure 2A,B for each of the analysed *SAMM50* polymorphisms, carriers of a *SAMM50* risk variant also carried the *PNPLA3* 148M risk variant in each cohort of liver disease in about 90% for rs3827385 and in far more than 90% for rs3761472. In addition, among patients with a *SAMM50* wildtype, the presence of a *PNPLA3* 148M risk variant doubled in patients with alcoholic HCC compared to controls without liver disease (Figure 2). Thus, the presence of a *SAMM50* risk allele is closely linked to the presence of a *PNPLA3* 148M risk allele, and the presence of a *PNPLA3* 148M allele also increases the risk for alcoholic HCC in carriers of a *SAMM50* wildtype. 

## 3. Patients and Methods

### 3.1. Patients

We included a cohort of *n* = 1060 patients with alcohol-associated cirrhosis, of whom *n* = 386 had HCC. Blood for DNA samples and clinical data of these patients was collected at the University Hospital Bonn, at the Berlin Department of Hepatology and Gastroenterology and at the Division of Hepatology of the Leipzig University Medical Centre. Furthermore, *n* = 134 patients with HCC due to HCV-induced cirrhosis from Bonn and Berlin, *n* = 237 healthy controls and *n* = 266 persons who misused alcohol without signs of significant liver disease were analysed. The healthy controls were collected among blood donors and participants in cancer screening programs. They were not known to suffer from liver disease and showed no signs thereof. Persons with alcohol misuse but without significant liver disease had been drinking a minimum of 60 g alcohol/day for women and 80 g alcohol/day for men over a period of at least 10 years without showing any sign of structural liver disease.

Cirrhosis was diagnosed by liver biopsy or on the basis of a combination of clinical, laboratory and radiological findings indicating cirrhosis. Standard demographic, clinical and laboratory data, such as age, sex, weight, presence of diabetes, ALT, AST, GGT, platelet count, bilirubin level and MELD score, were collected.

Patients were considered to suffer from alcohol-associated cirrhosis if average alcohol consumption above 300 g ethanol per week was reported and other causes of cirrhosis such as HBV or HCV infection or hemochromatosis were excluded.

Diagnosis of Hepatocellular carcinoma was made according to international guidelines [2] and based on histology if radiological findings were not unequivocal. 

All controls and patients were of Caucasian ethnicity. 

### 3.2. Methods

#### Determination of *SAMM50* and *PNPLA3* Genotypes

We extracted genomic DNA from 200 µL EDTA blood with the help of the QIAamp Blood Mini Kit (Qiagen, Hilden, Germany) following the manufacturer’s instructions. The different polymorphisms were determined by melting curve analysis. We used LightSNiP (SimpleProbe) assays from TIB-MolBiol (Berlin, Germany). In total, 1 µL of sample DNA was added to 0.5 µL of the LightSNiP reagent mix, 5 µL of Blue ProbeqPCR 2× Mix (Biozym Scientific GmbH, Hessisch Oldendorf, Germany) and 3.5 µL PCR grade water (Invitrogen, Paisley, UK). We performed real-time PCR on a LightCycler^®^ 96 system (Roche, Germany) as indicated by the protocol of the manufacturer. 

### 3.3. Statistical Analysis

IBM SPSS Statistics software version 28 (IBM, New York, NY, USA) was used for analysis of the data. We tested for significant deviations from the Hardy–Weinberg equilibrium by using an exact test (https://ihg.helmholtz-muenchen.de/cgi-bin/hw/hwa1.pl (accessed on 26 October 2022)). Genotypes were compared with the help of Pearson’s goodness-of-fit Chi^2^ test. Fisher’s exact test was used to compare qualitative data. Quantitative data were analysed by Student’s t-test for normally distributed or Wilcoxon–Mann–Whitney-U test for not normally distributed data. The clinical data of the different study cohorts were analysed by Analysis of Variance (ANOVA) with Bonferroni correction as post-hoc test. Univariate analysis followed by a multivariate forward binary regression was used after performing univariate analysis to test for independency of parameters associated with the risk for HCC with *p* < 0.05 for inclusion and *p* > 0.1 for exclusion of parameters. For testing potential linkage disequilibrium, we used a web-based application (https://zzz.bwh.harvard.edu/plink/ld.shtml#ld1 (accessed on 26 October 2022) and expectation-maximisation method. *p* < 0.05 was regarded as level of significance.

## 4. Discussion

In the present study, we analysed the contribution of two common *SAMM50* variants (rs3827385 and rs3761472) as risk factors for HCC in a large cohort of patients with alcohol-associated cirrhosis, with alcoholic HCC and appropriate control cohorts of healthy subjects, subjects with alcohol abuse but an absence of significant liver disease and patients with HCC due to viral hepatitis. Although carriers of each *SAMM50* minor allele showed an increased risk for HCC, this association was lost in multivariate analysis because the carriage of the well-known *PNPLA3* 148M risk variant is closely linked to the carriage of one of the investigated *SAMM50* variants and explains additional cases of HCC. Therefore, variation at the *PNPLA3* I148M locus seems a much better predictor for alcoholic HCC risk than the genetic variation of *SAMM50* rs3827385 and rs3761472.

It is known that only 10–20% of patients with alcohol abuse will develop alcohol-associated liver disease [25]. Genetic risk prediction might be helpful to identify patients in need of preventive measures and early screening for complications. Recently, Whitfield and colleagues developed a risk score for alcohol-associated cirrhosis analysing three large cohorts of patients [15]. Among eight genetic risk loci, only three became part of the final risk score, which included also the presence of diabetes as a clinical parameter. Of note, this risk score yielded higher values in patients with alcoholic HCC compared to patients with alcohol-associated cirrhosis. The three genetic parameters remaining in the final risk score were *TM6SF2* rs10401969, *HSD17B13* rs6834314 and *PNPLA3* rs738409. All three risk loci are not only established risk factors for alcohol-associated cirrhosis but also for alcohol-related HCC [7,8,10,26]. Rs738409 in *PNPLA3,* which results in an amino acid exchange at position 148 from isoleucine to methionine, proved superior to both investigated *SAMM50* variants in our analysis. 

In patients at risk for alcoholic HCC, the development of a genetic risk score might be particularly useful to tailor screening investigations, which are recommended by international guidelines for all patients at risk [2,3]. Because the genetic risk factors discovered so far are not sufficiently precise to define subgroups for screening in alcohol-associated cirrhosis [27], new genetic risk factors for HCC are investigated. Among others, a common variant in the *toll-like receptor 5*, rs5744174, was linked to the development of HCC [28], while genetic variation in platelet receptors does not seem to play a role [29]. In the same line, we analysed two polymorphisms in *SAMM50* because a recent genome-wide association study linked the genetic variation of *SAMM50* to the development of HCC [16]. 

*SAMM50* is a protein belonging to the outer membrane of mitochondria, which is necessary for the proper function of respiratory chain complexes [21]. Overexpression as well as the knock-down of *SAMM50* led to distorted mitochondrial morphology in human cell lines [30]. Interestingly, granzyme B-mediated apoptosis is facilitated by interaction with *SAMM50*, suggesting a role for *SAMM50* in cell death by reactive oxidative species and in lymphocyte-mediated cell death, e.g., of cancer cells [31,32]. In addition, *SAMM50* is involved in mitophagy, a process of mitochondrial degradation [33,34]. Since mitochondria play a key role in lipid metabolism [23] and non-alcoholic and alcoholic fatty liver disease are also marked by oxidative stress [23,25], a crucial involvement of *SAMM50* in the pathomechanism leading to fatty liver disease is conceivable.

However, the functional consequences of genetic variation of common *SAMM50* polymorphisms remain poorly understood. It was suggested that *SAMM50* is upregulated in patients with NAFLD, that this upregulation is impaired in the presence of genetic variants in polymorphisms associated with NAFLD and that experimental knock-down of *SAMM50* leads to intracellular triglyceride accumulation [18]. In line with changed expression, both rs3761472 and rs3827385 were reported to affect the binding of transcription factors and were linked to *SAMM50* mRNA expression level in adipose tissue [16]. 

Concerning the *PNPLA3* I148M variant, the exact molecular mechanisms leading to cirrhosis and HCC have not been established but the functional relevance was clarified in some aspects. However, experiments are hampered by the difficulty that simple inactivation or overexpression in mice does not mimic human disease. One important factor seems to be that *PNPLA3* 148M is more resistant to ubiquitylation and accumulates on lipid droplets [35], which leads to steatosis [36]. Using liver cell cultures derived from human pluripotent stem cells, the effect of the *PNPLA3* 148M variant causing a NAFLD phenotype was reproduced and linked to increased NF-kB and interleukin 6 activity [37]. Transduction of primary hepatocytes with the *PNPLA3* 148M variant resulted in the upregulation of CXC chemokines and created a tumorigenic milieu upon lipid stimulation [38], linking this genetic variant not only to NASH but also to HCC development. 

Several genetic risk loci for NAFLD are shared with alcohol-associated cirrhosis and alcoholic HCC. This holds true, among others, for *PNPLA3*, *TM6SF2* and *HSD17B13* [39]. Therefore, it would not be surprising if *SAMM50* had to be added to this list. While only one study has linked the genetic variation of *SAMM50* to HCC [16], the association to NAFLD was reported from different cohorts [17,18,40] and confirmed in a meta-analysis [41]. However, it is remarkable that all these studies included exclusively Asian patients, while the only study involving Caucasian patients was very small (40 cases and 24 controls) and did not perform multivariate analysis of the different genetic risk variants [42]. By contrast, in a study comprising a large multi-ethnic cohort of patients with the aim to develop a genetic risk score for NAFLD, genetic variation of *SAMM50* was only significantly associated with NAFLD in Japanese Americans and Latinos but not in Americans of Caucasian ethnicity or African Americans [20]. These differences indicate that *SAMM50* is not a universal genetic risk factor for NAFLD but is dependent on the genetic background of the patients. Still, the study by Wang et al., which linked both rs3827385 and rs3761472 *SAMM50* variants to HCC, did not comprise only Asian patients. However, on closer analysis, rs3761472 did not meet statistical significance in the initial cohort when patients with chronic liver disease were considered as controls, as would be most appropriate. For both polymorphisms, the association to HCC in the five validation cohorts is based mainly on one cohort with 100 cases of HCC on mixed background of liver disease aetiology and 214 controls. Therefore, diverging results in our study might be explained by differences in ethnical background, variation in the aetiology of HCC and differences in patient numbers.

Although we analysed a large, well-characterised and well-controlled cohort of patients, we cannot exclude that one of the investigated polymorphisms might be linked to HCC in a considerably larger dataset. However, if much more patients were needed to detect an association with HCC, we might assume that this genetic risk factor would not yield a predictive value for the majority of patients. Next, our study involved only Caucasian patients, so that genetic variation of *SAMM50* might still be associated with HCC independently from *PNPLA3* in Asian patients. Finally, although so far no other *SAMM50* polymorphisms apart from rs3827385 and rs3761472 have been linked to HCC, we cannot exclude the presence of such a polymorphism in *SAMM50*.

In summary, in a large cohort of Caucasian patients with alcoholic HCC, alcohol-associated cirrhosis and different controls, we did not find that genetic variation of the two *SAMM50* polymorphisms rs3827385 and rs3761472 previously reported as independent risk factors for HCC in NAFLD conferred an increased risk for HCC in addition to the presence of the *PNPLA3* 148M variant in patients with cirrhosis related to alcohol abuse. Until now, genetic variations of *SAMM50* rs3827385 and rs3761472 should not be used as biomarkers in Caucasian patients at risk for alcoholic HCC. 

## Figures and Tables

**Table 1:** Demographic, clinical and laboratory details on the study cohorts.

	Alcohol-Associated Cirrhosis		HCV HCC	Alcohol Misuse without Cirrhosis	Healthy Controls
	with HCC	without HCC			
Total number	386	674	134	266	237
Age, mean (range)	63.5 (36–87) ^a,b^	56.4 (27–92) ^c,d^	59.6 (38–82) ^d^	42.5 (18–81) ^e^	39.6 (20–75)
Sex (% male/female)	88.3/11.7 ^f^	70.6/29.4 ^g,h^	62.1/37.9 ^g^	85.4/14.6 ^i^	58.6/41.4
Bilirubin [mg/dL], (Mean ± SD)	2.78 ± 4.16 ^j^	4.00 ± 6.71 ^g^	2.63 ± 3.49	0.80 ± 1.22	n.d.
ALT [IU/L], (Mean ± SD)	49.2 ± 56.7	43.1 ± 110.0	71.9 ± 56.2	44.5 ± 49.9	n.d.
AST [IU/L], (Mean ± SD)	88.8 ± 109.8 ^k^	72.5 ± 168.5	85.9 ± 81.1	53.4 ± 65.4	n.d.
GGT [IU/L], (Mean ± SD)	273.5 ± 335.2 ^k,l^	197.1 ± 227.1	118.6 ± 127.0	203.4 ± 381.6	n.d.
Platelet count [* 10^3^/µL], (Mean ± SD)	150.9 ± 90.8 ^g^	152.3 ± 118.0 ^g^	111.4 ± 71.2 ^g^	229.3 ± 85.2	n.d.
MELD (Mean ± SD)	13.7 ± 6.8 ^n^	16.8 ± 7.2	15.2 ± 5.0	n.d.	n.d.
Diabetes (%)	46.4 ^m^	22.9	17.9	n.d.	n.d.

^a^ = *p* < 0.001 vs. cirrhosis without HCC, vs. alcoholic controls and vs. healthy controls; ^b^ = *p* < 0.05; ^c^ = *p* < 0.01 vs. HCV HCC; ^d^ = *p* < 0.001 vs. alcoholic controls and vs. healthy controls; ^e^ = *p* < 0.05; ^h^ = *p* < 0.01; ^i^ = *p* < 0.001 vs. healthy controls; ^f^ = *p* < 0.001 vs. cirrhosis without HCC, vs. HCV HCC and vs. healthy controls; ^g^ = *p* < 0.001; ^k^ = *p* < 0.05 vs. alcoholic controls; ^j^ = *p* < 0.01 vs. cirrhosis without HCC and vs. alcoholic controls; ^l^ = *p* < 0.01; ^m^ = *p* < 0.001 vs. cirrhosis without HCC and vs. HCV HCC; ^n^ = *p* < 0.001 vs. cirrhosis without HCC; Legend: ALT: alanine aminotransferase; AST: aspartate aminotransferase; GGT: Gamma glutamyl transferase; HCC hepatocellular carcinoma; MELD: model for end-stage liver disease; n.d. not done; Statistical analysis by ANOVA with Bonferroni correction as post-hoc test for quantitative data and Pearson’s goodness-of-fit Chi^2^ test qualitative data.

**Table 2:** Genotype distribution of the *SAMM50* and *PNPLA3* polymorphisms.

Genotype	Alcohol-Associated Cirrhosis (*n* = 1060)		HCV HCC (*n* = 134)	Alcohol Misuse without Cirrhosis (*n* = 266)	Healthy Controls (*n* = 237)
	with HCC *n* = 386	without HCC *n* = 674			
*SAMM50* rs3827385					
TT	162 (42.0%)	381 (56.5%)	84 (62.7%)	176 (66.2%)	160 (67.5%)
TC	178 (46.1%) ^a,d,g^	246 (36.5%) ^b^	40 (29.9%)	84 (31.6%)	65 (27.4%)
CC	46 (11.9%) ^a,d,f^	47 (7.0%) ^e^	10 (7.5%)	6 (2.3%)	12 (5.1%)
allele frequency T/C	65.0%/35.0% ^a,d,g^	74.8%/25.2% ^b^	81.2%/18.8%	82.0%/18.0%	81.2%/18.8%
*SAMM50* rs3761472					
AA	158 (40.9%)	374 (55.5%)	87 (64.9%)	180 (67.7%)	157 (66.2%)
AG	179 (46.4%) ^a,d,g^	248 (36.8%) ^c,f^	38 (28.4%)	77 (28.9%)	67 (28.3%)
GG	49 (12.7%) ^a,d,g^	52 (7.7%)	9 (6.7%)	9 (3.4%)	13 (5.5%)
allele frequency A/G	64.1%/35.9% ^a,d,g^	73.9%/26.1% ^c^	79.1%/20.9%	82.1%/17.9%	80.4%/19.6%
*PNPLA3* rs738409					
CC	96 (24.9%)	280 (41.5%)	64 (47.8%)	146 (54.9%)	129 (54.4%)
GC	199 (51.6%) ^a,d,g^	306 (45.4%) ^c^	54 (40.3%)	104 (39.1%)	89 (37.6%)
GG	91 (23.6%) ^a,d,g^	88 (13.1%) ^c^	16 (11.9%)	16 (6.0%)	19 (8.0%)
allele frequency C/G	50.6%/49.4% ^a,d,g^	64.2%/35.8% ^d^	67.9%/32.1%	74.4%/25.6%	73.2%/26.8%

^a^ = *p* < 0.001 vs. cirrhosis without HCC; ^b^ = *p* < 0.05; ^c^ = *p* < 0.01; ^d^ = *p* < 0.001 vs. alcoholic controls and vs. healthy controls; ^e^ = *p* < 0.01 vs. alcoholic controls; ^f^ = *p* < 0.05; ^g^ = *p* < 0.001 vs. viral HCC; Legend: HCC hepatocellular carcinoma; *SAMM50*: Sorting and assembly machinery component 50 homolog; *PNPLA3*: Petaton-like phospholipase domain-containing protein 3.

**Table 3:** Regression Analysis for possible HCC risk factors.

Univariate Analysis				
			95% CI	
Parameter	P	OR	Lower	Upper
Age	4.0 × 10^−24^	1.086	1.069	1.103
Sex (male)	1.1 × 10^−9^	3.151	2.178	4.559
Diabetes	2.4 × 10^−13^	2.909	2.186	3.871
*PNPLA3* 148 (IM/MM)	6.4 × 10^−8^	2.147	1.627	2.832
rs3827385 (TC/CC)	6.0 × 10^−6^	1.798	1.396	2.316
rs3761472 (AG/GG)	6.0 × 10^−6^	1.799	1.396	2.318
Multivariate Analysis *				
			**95% CI**	
**Parameter**	**P**	**OR**	**Lower**	**Upper**
Age	1.2 × 10^−16^	1.078	1.059	1.097
Sex (male)	8.1 × 10^−8^	3.174	2.081	4.840
Diabetes	6.3 × 10^−5^	1.930	1.399	2.662
*PNPLA3* 148M	1.6 × 10^−5^	2.083	1.492	2.909

Legend: * (including all significant parameters from the univariate analysis); CI, confidence interval; OR, odds ratio; HCC: hepatocellular carcinoma; *PNPLA3*: Patatin-like phospholipase domain-containing protein 3.

## Data Availability

Data available on request due to privacy restrictions.

## Abbreviations

ANOVA	Analysis of Variance
ALT	alanine aminotransferase
AST	aspartate aminotransferase
HCC	hepatocellular carcinoma
HCV	hepatitis C virus
*HSD17B13*	17β-Hydroxysteroid dehydrogenase type 13
GGT	Gamma-glutamyl transferase
MAF	minor allele frequency
MELD	Model for End-Stage Liver Disease
NAFLD	non-alcoholic fatty liver disease
NASH	non-alcoholic steatohepatitis
NF-kB	nuclear factor ‘kappa-light-chain-enhancer’ of activated B-cells
*PNPLA3*	patatin-like phospholipase domain-containing protein 3
OR	odds ratio
*SAMM50*	Sorting and assembly machinery component 50 homolog
TLR	toll-like receptor
*TM6SF2*	Transmembrane 6 superfamily 2
